# Beat osteoporosis — nourish and exercise skeletons (BONES): a group randomized controlled trial in children

**DOI:** 10.1186/s12887-020-1964-y

**Published:** 2020-02-21

**Authors:** Christina D. Economos, Erin Hennessy, Kenneth Chui, Johanna Dwyer, Lori Marcotte, Aviva Must, Elena N. Naumova, Jeanne Goldberg

**Affiliations:** 1 Friedman School of Nutrition Science and Policy, Tufts University, 150 Harrison Avenue, Boston, MA 02111 USA; 2 Tufts University School of Medicine, Boston, MA USA; 3 Frances Stern Nutrition Center, Tufts Medical Center, Boston, MA USA; 4 Jean Mayer USDA Human Nutrition Research Center on Aging and Tufts University School of Medicine, Boston, MA USA; 5 Office of Dietary Supplements, National Institutes of Health, Bethesda, MD USA; 6 Boston Public Schools, Boston, MA USA

**Keywords:** Bone, Randomized controlled trial, Osteoporosis, Weight loading physical activity, Calcium, Child, Health behavior

## Abstract

**Background:**

Lifelong healthy habits developed during childhood may prevent chronic diseases in adulthood. Interventions to promote these habits must begin early. The BONES (Beat Osteoporosis – Nourish and Exercise Skeletons) project assessed whether early elementary school children participating in a multifaceted health behavior change, after-school based intervention would improve bone quality and muscular strength and engage in more bone-strengthening behaviors.

**Methods:**

The 2-year BONES (B) intervention included bone-strengthening physical activity (85 min/week), educational materials (2 days/week), and daily calcium-rich snacks (380 mg calcium/day) delivered by after-school program leaders. BONES plus Parent (B + P) included an additional parent education component. From 1999 to 2004, *n* = 83 after-school programs (*N* = 1434 children aged 6–9 years) in Massachusetts and Rhode Island participated in a group randomized trial with two intervention arms (B only, *n* = 25 programs; B + P, *n* = 33) and a control arm (C, n = 25). Outcome measures (primary: bone quality (stiffness index of the calcaneus) and muscular strength (grip strength and vertical jump); secondary: bone-strengthening behaviors (calcium-rich food knowledge, preference, and intake; and physical activity level (metabolic equivalent time (MET) score, and weight-bearing factor (WBF) score)) were recorded at baseline, and after years one and two. Analyses followed an intent-to-treat protocol, and focused on individual subjects’ trajectories along the three time points adjusting for baseline age and race via a mixed-effects regression framework. Analyses were performed with and without sex stratification.

**Results:**

Children in B + P increased bone stiffness compared to C (*p* = 0.05); No significant changes were observed in muscle strength, food knowledge, or vertical jump. Children in B + P showed significant improvement in their MET and WBF scores compared to C (*p* < 0.01) with a stronger effect in boys in both B and B + P (all p < 0.01).

**Conclusion:**

After-school programs, coupled with parental engagement, serving early elementary school children are a potentially feasible platform to deliver bone-strengthening behaviors to prevent osteoporosis in adulthood, with some encouraging bone and physical activity outcomes.

**Trial registration:**

ClinicalTrials.gov NCT00065247.

Retrospectively registered.

First posted July 22, 2003.

## Introduction

Childhood is a crucial period of social, cognitive, and physiological development [1]. Habits acquired then are often sustained throughout the lifespan [2], emphasizing the need to engage children with healthy behaviors early in life. Children who meet recommendations for physical activity and appropriate nutrition tend to have stronger bones, better cardiovascular health, and exhibit better academic performance and higher self-esteem [3, 4]. Unfortunately, American children today spend less time in physical activity and consume inadequate amounts of key nutrients which could impede their growth and development [5–7]. Therefore, it is worthwhile to develop evidence-based programs that engage children in health behaviors that will encourage a strong foundation for adulthood.

Among the many health behaviors that are critical during childhood are weight-bearing physical activity and calcium consumption. These behaviors independently and synergistically contribute to bone mass accrual which is critical for strong skeletal development [8, 9]. Peak bone mass is reached by most individuals during adolescence, and low accumulation of bone mineral during pre-pubertal years increases risk of fractures and the porous and weak bones which are hallmarks of osteoporosis later in life [10]. To mitigate this risk, the Surgeon General’s Report on Bone Health and Osteoporosis suggested teaching youth healthy bone-building behaviors that can be incorporated into children’s daily routines [11].

While much research supports this recommendation for early intervention, few multi-component, bone-strengthening intervention trials have been developed for children. Previous attempts have mostly targeted children of older ages (9–16 years) for durations of less than 1 year with a focus on increasing either calcium intake or bone-strengthening physical activity delivered via school-based programs [12–15]. The few interventions outside of the school environment have been conducted largely on older girls or in laboratory, rather than in real-world settings [16–19], and there are few longitudinal community-based studies of bone quality in children [20, 21].

Alternatively, community-based settings that can reach large numbers of younger children of both sexes with multiple intervention components need to be explored. In particular, after-school programs present a unique and promising opportunity to reach a younger, more diverse sample of boys and girls [22]. Currently, over 10 million children participate in one or more after-school programs, demonstrating the potential and widespread reach of intervening through this unique platform [23]. Since these programs have fewer requirements for curriculum and scheduling compared to schools, they provide greater opportunity to deliver multifaceted interventions. The BONES Project addressed the current paucity of bone-building interventions for young children, using after-school programs as an intervention site. The primary aims of the BONES Project were (1) to increase the bone quality and muscular strength of children participating in the intervention; and (2) to improve knowledge and level of bone health and behaviors (e.g., level of bone-strengthening physical activity and calcium intake). This article presents the outcomes from the two-year intervention targeting bone-strengthening physical activity and dietary behaviors of early elementary school children attending after-school programs in the Northeastern U.S.

## Methods

### Study design

The BONES (Beat Osteoporosis – Nourish and Exercise Skeletons) project was a community-based, group-randomized, controlled trial conducted from 1999 to 2004. It was designed to test the feasibility of influencing bone health in early elementary school children by modifying health behavior through the introduction of bone-strengthening physical activity, education on nutrition and bone health, and the delivery of calcium-rich snacks in after-school programs. A three-arm design allowed the impact of the main BONES intervention (B) and an enhanced BONES intervention which contained a parental/caregiver component (B + P) to be assessed against a control group (C). A group-randomized trial design was used to test the hypothesis that children attending the intervention programs (B and B + P) would exhibit greater bone quality and muscle strength, and more bone-strengthening behaviors over a two-year period than children attending an after-school program without the intervention [24].

This study was reviewed by, approved, and adhered to all procedures outlined by the Tufts University Institutional Review Board and the National Institutes of Health. Written consent was obtained from all parents/guardians of participating children, prior to the start of the intervention.

### Recruitment and setting

The target population was young elementary school children between the ages of 6-to-9 years old attending after-school programs. To reach this population, we first compiled a systematic profile of communities in Massachusetts and Rhode Island including key community characteristics: number of elementary schools, percentage of children eligible for free or reduced-price meals, and racial-ethnic diversity. Lower income communities (based on the percent of children eligible for free or reduced-price meals) that had multiple after-school programs (3 or more per community) with > 40 eligible children per program were considered eligible.

Within the 33 eligible communities, we identified 384 after-school programs for potential participation by contacting school superintendents or after-school program directors directly to screen for interest in study participation. Of those programs contacted, 181 did not respond, did not contain an after-school program, or were uninterested in participating. For all others, an informational packet which contained a description of the BONES Project was sent to the school superintendent and/or after-school program director along with a letter inviting the school district or program to participate. Mailings were followed by phone calls and when appropriate, a meeting was arranged at which the researchers presented an overview of the projected after-school program. Following these informational meetings, an additional 60 programs were excluded based on lack of interest, program structure, or administrative turnover. Researchers visited the remaining 143 individual program sites to discuss the program in more detail with site leaders and to obtain information about program structure. The informational packet, initial presentation, and individual site visits represented a comprehensive procedure that facilitated commitments from school superintendents and program directors. Letters of agreement were developed and signed by the programs to serve as a formal commitment (*n* = 83, after-school programs) (Fig. 2).

Once after-school programs agreed to participate, program staff members were trained on procedures and strategies to recruit families using written materials and flyers in three languages (English, Spanish and Portuguese). For accuracy of translation and to ensure that all participants received identical information, all translated materials were back-translated into English by a different individual and revised, accordingly. All children between the ages of 6-to-9 years who attended the after-school program were eligible to enroll upon written consent of the parent/guardian. Once an after-school program recruited a minimum of 8 children, it was randomized in a 2:1 ratio into an intervention (B or B + P) or control (C) group. During the randomization process we considered a blocked design, in which the size of the after-school program and community socioeconomic status [25] were balanced in a manner that the final three groups, across all communities, had a similar number of programs, number of participants per program, and a similar SES distribution. Control programs were eligible to receive the curriculum materials upon conclusion of the intervention period if they wished. Ultimately, 25 programs (469 children) were randomized to the BONES intervention, 33 programs (611 children) were randomized to the BONES + Parent intervention, and 25 programs (254 children) were randomized to the control.

### Intervention

#### Theoretical framework

**Fig. 1:**
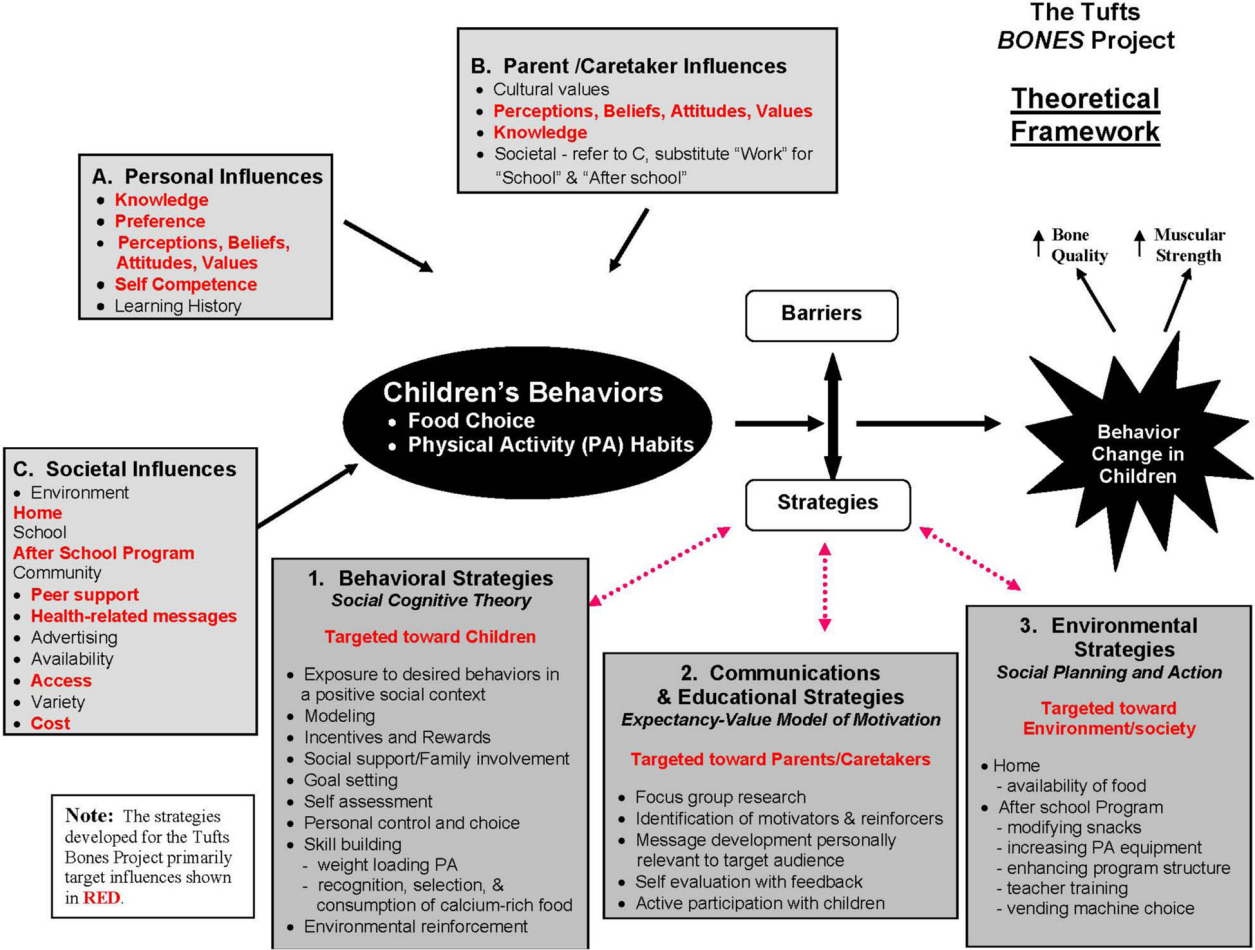
The BONES Project theoretical framework. The theoretical framework integrated existing constructs and prior research to identify (1) Behavioral; (2) Communications and Educational; and (3) Environmental behavior change strategies to influence (**a**) personal; (**b**) parental/caregiver, and (**c**) societal influences on child health behavior. Influencing factors szhown in red were targeted by the intervention

The BONES Project theoretical framework (Fig. 1) combines elements from the Expectancy-Value Model of Motivation [26], which uses the Health Belief Model [27] and the Theory of Reasoned Action [28], Social Cognitive Theory [29], and the Social Planning and Action Model [26, 30]. This illustrates how the factors that influence three types of behavior change strategies (behavioral; communications and educational; and environmental) interact.

#### Components

The two intervention groups (B and B + P) received a comprehensive, three-component curriculum for 20 weeks per year for 2 years. The development of the project components was informed by formative research with focus groups and a six-week pilot study [31]. The intervention consisted of the following components: (1) *Let’s Eat:* calcium-rich snacks which offered children an average of 380 mg of calcium per day; (2) *Let’s Play:* active games which provided 20 min of vigorous activity 3 days per week with a 5 min jumping component that enabled ground reaction forces between 4 and 7 times body weight, implemented 5 days per week; and (3) *Let’s Explore:* nutrition education lessons delivered in a fun, hands-on manner 2 days per week. The curriculum was designed to fit within the structure of various after-school programs, which typically offer homework and academic assistance and recreational activities and snacks, rather than formal physical activity programming. The intervention program groups received additional physical activity equipment to help implement component 2, *Let’s Play*. The B + P group received all three curriculum components as well as an additional (4) parent/caretaker outreach component sent home (e.g. newsletters to complement lessons, educational worksheets, coupons, and a detailed community directory/resource guide for family-friendly active living and healthy eating). The intervention group after-school program staff attended comprehensive training programs at the start of each intervention year and also received ongoing support from study research staff in the form of newsletters, site visits, and phone calls.

#### Process evaluation

The BONES process evaluation assessed both dose (the amount of time research participants spent engaged in the program), and fidelity (the extent to which the intervention was delivered according to the intended delivery) of the program. After-school program leaders were provided with a daily attendance sheet to track child participation in each of the three program components. For example, program leaders tracked the lesson conducted for *Let’s Explore*, the activity performed for *Let’s Play*, and the calcium-rich snack offered for *Let’s Eat*. After-school programs were also evaluated for program fidelity through bi-yearly direct observations (site visits) as well as year-end surveys of after-school programs. These data were compiled into two compliance measurements: (i) the number of evaluation/attendance forms returned by after-school programs in year 1 and 2; and (ii) research study staff’s perceived fidelity to the intervention by after-school programmatic staff. The percentage of programs returning evaluation/attendance sheets at the end of years 1 and 2 ranged from 88–91% and 72–85%, respectively, and perceived fidelity ranged from 1.9–2.1 over the 2 years (on a 1–3 scale: 1 = good/excellent, 2 = okay, but inconsistent, and 3 = poor, did not do). This information was combined to construct an implementation score to rank programs (low, medium, high) for their dose and fidelity to the intervention. Since intervention compliance did not differ by assignment and was not consistently associated with outcomes, these detailed data are not presented.

### Outcomes

The primary outcomes were bone quality and muscular strength. Bone quality is defined operationally as a composite of factors that help bones to resist fracture [32] and was measured using bone stiffness index (SI) (%) of the calcaneus. Secondary outcomes included body composition (BMI and percent body fat), and knowledge and level of bone-strengthening behaviors (preference for and consumption of calcium-rich foods and physical activity level). All measurements were obtained in the field at the after-school program by trained research staff. Subjects were measured at baseline in the fall/early winter of the first year with follow up measures each spring (years 1 and 2). The testing day was organized like a health-fair for the children and they each received a prize at the end of the day for their participation.

#### Bone quality

Broadband ultrasound attenuation (BUA) and ultrasound velocity or speed of sound (SOS) of the calcaneus were measured in the field. Measurements were obtained using a calcaneal quantitative ultrasound (QUS) device (Lunar Achilles +, GE Medical, Milwaukee, WI) which is reliable and valid [33], small, portable, inexpensive, and approved by the Food and Drug Administration (FDA). Two to three consecutive measurements with repositioning were performed following the manufacturer protocol [34]. A linear combination of BUA and SOS was used to calculate bone stiffness index (SI) (%) of the calcaneus by the formula (0.67 × BUA + 0.28 × SOS) which was evaluated as the outcome of interest.

#### Muscular strength: grip strength and vertical jump

Grip strength was measured with a Smedley III Hand Dynamometer (Country Technology Inc., Gay Mills, WI) following manufacturer protocol [35]. Three trials with each hand were performed, adjusting for grip size, alternating hands, and with a 15-s rest between each trial to avoid excessive fatigue. The highest result for each hand (recorded to the nearest 0.1 kg) was recorded, and the dominant hand was noted. Vertical jump distance was used to assess lower body strength and explosive power. The Just Jump System (Probotics, Huntsville, AL) consisted of a computerized rubber mat that converts hang time into a linear measure of vertical jump height and records the results in inches. Three trials were performed and the child’s maximum vertical jump height was used in analyses [36].

#### Body composition: BMI and percent body fat

Height and weight were measured without shoes, by trained study staff, in triplicate (or until three measurements were within  ±0.25 cm and 0.5 lb., respectively), and averaged. Height was measured to the nearest 0.1 cm using a portable stadiometer (Seca model 214) and weight was measured in light clothing to the nearest 0.5 lb. (SECA model 812) on a digital scale following standard procedures [37]. Body Mass Index (BMI) was calculated as average body weight in kilograms divided by average height in meters squared (kg/m^2^). BMI z-score was then calculated based on the CDC Reference Growth Chart [38]. Body fat was calculated based on skinfold thickness measurements of the triceps and calf taken on the right side using standard protocol with Lange Skinfold Calipers (Beta Technology Inc.) which are accurate to  ±1 mm [39]. Percent body fat was calculated according to the age- and sex-specific prediction equations of Slaughter [40].

#### Knowledge and level of bone-health behaviors: nutrition and physical activity

Calcium-rich food intake, preference for them, and related nutrition knowledge. A checklist of calcium-containing foods was created and tested for validity and reliability for the study [41]. This tool allows assessment of calcium and dairy intake over a 24-h period. A pictorial survey of various foods was created for the study, modeled from work by Edmunds and Ziebland [42], and used to evaluate preference for and knowledge of calcium-rich foods. All assessments were based on child responses to each measurement tool during interviewer-assisted assessments. Children were given 11 pictures of child friendly foods; 5 were calcium-rich foods and 6 non-calcium-rich foods. For food preference, children sorted the food pictures into four groups: ‘likes a lot,’ ‘is okay,’ ‘don’t like,’ and ‘never tasted.’ For knowledge, the same pictures were sorted into three groups: ‘makes bones strong,’ ‘does not make bones strong,’ and ‘don’t know.’

Reported physical activity level and knowledge*.* A pictorial physical activity survey that assesses children’s physical activity levels and knowledge of bone-strengthening activities was created for the study and has been shown to be valid and reliable (Spearman’s *r* range for MET and WBF: 0.57–0.74, all *p* < 0.001) [43]. This tool allows assessment of physical activity level and intensity expressed as a MET (metabolic equivalent time) score and a WBF (weight-bearing factor) score. All assessments were based on child responses to each measurement tool during interviewer-assisted assessments. For knowledge, children were given 10 pictures with child-friendly activities; 6 of medium-high impact activity and 4 common activities with low impact. Children sorted the pictures into three groups: ‘makes bones strong,’ ‘does not make bones strong,’ and ‘don’t know.’

#### Other health/medical information

At baseline, a comprehensive 70-item health questionnaire was mailed to parents with a postage paid, pre-addressed return envelope. Questions included medical history items, sociodemographic information (e.g., parent education level, age, and race/ethnicity), child activity (including sports and lesson involvement), dietary restrictions, and parenting practices related to diet and screen time. A questionnaire with Tanner stage items [44] was sent to parents/caregivers at the conclusion of the intervention.

### Sample size estimation

The study was powered to detect an estimated difference between groups of 0.22 bone stiffness index (SI) (or 5%) based on a standard deviation of the difference of 1.50 stiffness units. This resulted in the need for 261 subjects per group, to test the difference in groups at α = 0.05 and 80% power. To account for both clustering within the after-school program and a 36% attrition rate over the study period, a sample size of 377 subjects in each group was estimated, based on the only published data available at the time [45].

### Statistical analyses

#### Descriptive statistics

The demographic variables, primary outcomes, and secondary outcomes at each time point were tabulated by program arm and sex. Descriptive statistics including number of respondents, means, and 95% confidence intervals were compiled and tabulated.

#### Regression analysis

We employed a linear mixed effects model approach to estimate the rates of change in the suggested outcomes overtime [46]. The general model is:

1 $$ {Y}_{ij}={\beta}_0+{\beta}_1\ {arms}_i\times {timei}_j+{\beta}_2\  race/{ethnicity}_i+{\beta}_3\  baseline\ {age}_i+{\varepsilon}_{ij},\dots $$

where *Y*_*ij*_ represents the measurements of *i*^*th*^ participant at *j*^*th*^ time point, *arms* represents the three interventions (B: BONES, B + P: BONES + Parental/caregiver component, and C: Control), *time* is a continuous predictor indicating number of years since the start of the intervention (0: Baseline, 0.5: Post-intervention, and 1.5: Follow-up), *race/ethnicity* is a 4-level categorical variable representing white, black, Hispanic, and others, and *baseline age* is the age of the participant centered at 7 (the average age at baseline). While race/ethnicity and baseline age did not differ across programs, we adjusted for these variables with the intention to improve the precision of the regression models. We allowed for the slope of each intervention group to vary by specifying them as random cluster effects. Because the participants were randomized by after-school program, we also specified the random effects to be at both individual level and individual nested within after-school program to control for the correlated error within the child along time and the clustering between the programs. We applied a multi-level modeling technique (PROC MIXED) to allow for clustering at the individual due to repeated measurement, and clustering at the school level due to group-randomization, so that the variance in the individual level was computed at the school level first and then over the population.

#### Statistical tests of the research questions

We compared the difference between the three slopes (B, B + P, and C) captured by the coefficient *β*_*1*_ in equation (i). We tested the hypothesis that the trajectories of the outcomes would be different among the three groups, with the beneficial effects highest in B + P, followed by B, and C. Statistical analyses were first performed for all subjects, and then stratified by sex. Due to a smaller than expected sample of children that completed bone quality measurements, bone stiffness data were analyzed in two ways: with the two interventions arms, B and B + P, pooled together and independently. Two sub-analyses were performed as follows. First in order to identify whether the intervention benefits participants with and without low calcium intake differently, the change by sex and calcium status according to the guidelines at the start of the intervention (< 500 mg for children 1-to-8 years old; < 1300 mg for children > 8 years old) was evaluated and results are presented. Second, in order to identify whether the intervention benefits participants who may have received a higher intervention dose compared to those who received a lower dose, the change in all outcomes was evaluated. Because no differences were identified according to implementation dose, those results are not presented.

SAS 9.2 PROC MIXED was used for the analysis. Statistical significance was based on an alpha-level of 0.05.

## Results

### Participant flow and recruitment

Recruitment in the Winter-Spring of 1999–2000, was less than projected; only 46 programs were enrolled (*N* = 810 subjects). There was a mean of 17 participants per after-school program. Therefore, to obtain the required sample size, a second round of recruitment was carried out during the Spring-Summer of 2001 (*n* = 37 programs, *N* = 624 participants), also with a mean of 17 participants per after-school program. After-school programs were located in schools (42%), YMCA’s (18%), community agencies (18%), Boys and Girls Clubs (15%), and other private agencies (7%).

**Fig. 2:**
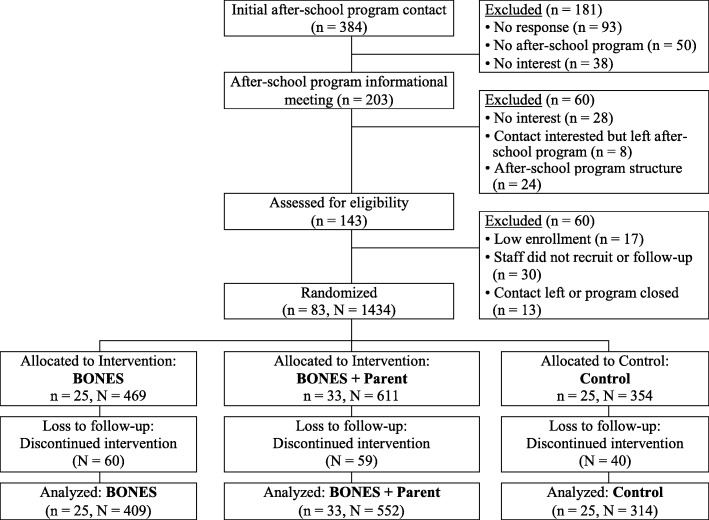
Flow diagram of recruitment and analysis in the BONES Project. Note: n = number of after-school programs; N = number of children

All participants received the two-year intervention as designed. A total of 83 after-school programs (*N* = 1434 participants) were randomized at baseline (Fig. 2). As detailed in Fig. 2, lack of participation was primarily due to lack of response to initial inquiries or lack of interest/follow-up by the program (53%), structural limitations (26%), or administrative turnover in which the initial contact at a site expressed an interest that did not transfer to their successor (17%).

### Baseline characteristics and longitudinal outcomes

The baseline age, height, and weight and the three longitudinal measurements of the outcomes (T0, T1, T2) are displayed in Tables 1 and 2. Children that were randomized but never attended any testing days were excluded from analyses (*n* = 159). The demographic and outcomes data at baseline (T0) are similar across the three treatments groups in both sexes. Small but statistically significant mean differences did exist in baseline height and weight: males in the control group (C) were shorter and lighter than those in B or B + P groups. Females in the C group were lighter than those in the B group but with similar mean weight when compared to the B + P group. However, the BMI z-score was not significantly different among groups. No significant differences were observed between children who completed measurements at all three time-points compared to those completing only one or two.

**Table 1:** Descriptive statistics for male participants’ characteristics and outcomes by time point and intervention status (*n* = 641)

Variable	Time point	Control (C) *n* = 173			BONES (B) *n* = 207			BONES + Parents (B + P) *n* = 261		
		n	Mean	95% CI	n	Mean	95% CI	n	Mean	95% CI
Age (yr)	T0	143	7.16	(7.06, 7.27)	163	7.19	(7.06, 7.31)	210	7.19	(7.08, 7.3)
Height (cm)^a^	T0	133	121.35	(120.24, 122.45)	160	123.32	(122.36, 124.28)	205	123.33	(122.43, 124.24)
Weight (kg)^b^	T0	133	25.16	(24.26, 26.07)	160	27.00	(26.04, 27.95)	207	26.83	(25.98, 27.67)
Race/ethnicity, n (%)										
White	T0	72		(63.2%)	83		(64.3%)	118		(65.2%)
Black	T0	16		(14.0%)	8		(6.2%)	19		(10.5%)
Hispanic	T0	10		(8.8%)	13		(10.1%)	16		(8.8%)
Other	T0	16		(14.0%)	25		(19.4%)	28		(15.5%)
BMI z-score	T0	130	0.53	(0.36, 0.71)	158	0.76	(0.61, 0.91)	200	0.73	(0.59, 0.87)
	T1	102	0.64	(0.45, 0.84)	122	0.73	(0.57, 0.89)	154	0.74	(0.59, 0.89)
	T2	70	0.77	(0.53, 1)	76	0.90	(0.69, 1.11)	103	0.70	(0.5, 0.89)
Percent body fat (%)	T0	135	16.73	(15.66, 17.8)	159	16.12	(15.14, 17.11)	205	16.24	(15.33, 17.15)
	T1	106	16.55	(15.32, 17.78)	147	17.47	(16.4, 18.53)	158	17.48	(16.34, 18.61)
	T2	82	17.22	(15.55, 18.88)	74	20.37	(18.4, 22.34)	105	18.96	(17.51, 20.42)
Calcium-rich food knowledge score^c^	T0	134	6.35	(6.09, 6.61)	153	6.71	(6.42, 6.99)	203	6.73	(6.5, 6.96)
	T1	105	6.75	(6.46, 7.04)	142	7.11	(6.85, 7.37)	161	7.27	(7.02, 7.52)
	T2	80	6.96	(6.61, 7.32)	71	7.89	(7.53, 8.25)	103	7.63	(7.36, 7.9)
Physical activity knowledge score^c^	T0	132	6.08	(5.78, 6.38)	158	6.12	(5.85, 6.39)	207	6.11	(5.9, 6.31)
	T1	107	6.21	(5.93, 6.48)	145	6.54	(6.3, 6.79)	164	6.71	(6.5, 6.92)
	T2	83	6.37	(6.1, 6.65)	75	6.75	(6.48, 7.02)	106	6.83	(6.56, 7.1)
Calcium-rich food preference score^c^	T0	134	3.51	(3.3, 3.71)	152	3.53	(3.35, 3.72)	202	3.44	(3.27, 3.61)
	T1	105	3.41	(3.16, 3.66)	142	3.64	(3.45, 3.84)	161	3.54	(3.35, 3.73)
	T2	80	3.39	(3.13, 3.64)	71	3.52	(3.27, 3.78)	103	3.59	(3.37, 3.81)
Total calcium intake (mg)^c^	T0	122	1024	(923, 1125)	146	1077	(975, 1179)	198	1088	(1016, 1160)
	T1	101	952	(851, 1052)	142	1145	(1042, 1249)	159	1099	(1011, 1187)
	T2	81	1055	(935, 1174)	72	1042	(899, 1184)	105	1048	(946, 1150)
MET score^c^	T0	132	25.20	(23.18, 27.21)	158	24.90	(23.1, 26.7)	207	24.61	(23.05, 26.18)
	T1	107	23.70	(21.61, 25.79)	146	25.69	(23.87, 27.52)	164	25.57	(23.78, 27.37)
	T2	83	22.07	(19.76, 24.38)	75	26.46	(24.11, 28.82)	106	25.54	(23.52, 27.56)
WBF score^c^	T0	132	3.83	(3.45, 4.21)	158	3.75	(3.41, 4.1)	206	3.69	(3.39, 4)
	T1	107	3.56	(3.15, 3.96)	146	3.84	(3.47, 4.21)	164	3.93	(3.57, 4.3)
	T2	83	3.25	(2.78, 3.71)	75	4.23	(3.77, 4.7)	106	4.18	(3.77, 4.59)
Grip strength (kg)	T0	136	11.43	(11, 11.87)	160	11.55	(11.17, 11.94)	206	12.05	(11.66, 12.45)
	T1	107	11.21	(10.75, 11.68)	147	11.69	(11.26, 12.11)	163	12.34	(11.84, 12.84)
	T2	81	13.11	(12.38, 13.84)	77	13.23	(12.55, 13.92)	105	13.45	(12.79, 14.1)
Vertical jump (in)	T0	136	12.08	(11.7, 12.45)	158	12.40	(12.08, 12.72)	204	12.06	(11.8, 12.32)
	T1	105	12.39	(12.01, 12.78)	148	12.30	(11.99, 12.62)	162	12.63	(12.31, 12.95)
	T2	80	13.00	(12.54, 13.45)	76	12.86	(12.38, 13.34)	106	12.97	(12.53, 13.4)
Bone stiffness index	T0	57	75.03	(69.85, 80.21)	53	72.62	(68.63, 76.6)	76	73.12	(69.67, 76.58)
	T1	58	67.71	(63.51, 71.91)	84	69.66	(66.44, 72.88)	107	70.34	(67.69, 72.99)
	T2	57	68.41	(65.14, 71.68)	40	69.57	(65.1, 74.05)	75	71.11	(67.71, 74.5)

*Abbreviations*: *BMI* body mass index, *MET* metabolic equivalent time, *WBF* weight-bearing factor

^a^Average height of C is significantly lower than B and B + P

^b^Average weight of C is significantly lower than B and B + P

^c^Range of scores: Calcium-rich food knowledge score: 0–11; Physical activity knowledge score: 0–9; Calcium-rich food preference score: 0–5; Total calcium intake (mg): 0–3661.5; MET score: 0–46.8; WBF score: 0–7.5

**Table 2:** Descriptive statistics for female participants’ characteristics and outcomes by time point and intervention status (*n* = 634)

Variable	Time point	Control (C) *n* = 141			BONES (B) *n* = 202			BONES + Parents (P) *n* = 291		
		n	Mean	95% CI	n	Mean	95% CI	n	Mean	95% CI
Age (yr)	T0	111	7.1	(6.98, 7.21)	156	7.21	(7.09, 7.32)	221	7.17	(7.07, 7.27)
Height (cm)	T0	107	122.01	(120.68, 123.33)	152	123.10	(121.9, 124.29)	210	121.89	(120.95, 122.83)
Weight (kg)^a^	T0	107	25.24	(24.23, 26.25)	152	27.59	(26.4, 28.79)	211	26.19	(25.32, 27.06)
Race/ethnicity, n (%)										
White	T0	54		(64.3%)	69		(50.7%)	123		(67.2%)
Black	T0	8		(9.5%)	13		(9.6%)	18		(9.8%)
Hispanic	T0	4		(4.8%)	24		(17.7%)	18		(9.8%)
Other	T0	18		(21.4%)	30		(22.1%)	24		(13.1%)
BMI z-score	T0	101	0.50	(0.32, 0.68)	147	0.79	(0.63, 0.96)	207	0.66	(0.54, 0.78)
	T1	87	0.60	(0.38, 0.82)	114	0.68	(0.5, 0.87)	204	0.65	(0.53, 0.78)
	T2	60	0.69	(0.42, 0.96)	65	0.88	(0.63, 1.13)	146	0.73	(0.58, 0.89)
Percent body fat (%)	T0	109	19.66	(18.79, 20.53)	152	19.53	(18.53, 20.53)	207	19.77	(18.98, 20.56)
	T1	88	20.93	(19.51, 22.36)	136	21.07	(20.04, 22.1)	210	20.97	(20.07, 21.86)
	T2	60	21.21	(19.69, 22.74)	62	22.65	(21.06, 24.25)	150	21.89	(20.94, 22.85)
Calcium-rich food knowledge score^c^	T0	104	6.48	(6.16, 6.8)	152	6.47	(6.21, 6.74)	212	6.65	(6.44, 6.86)
	T1	89	6.67	(6.37, 6.98)	139	7.26	(7.03, 7.49)	206	7.31	(7.1, 7.52)
	T2	62	6.98	(6.61, 7.35)	65	7.72	(7.47, 7.98)	143	7.60	(7.38, 7.82)
Physical activity knowledge score^c^	T0	110	5.76	(5.44, 6.09)	155	5.85	(5.57, 6.13)	211	6.04	(5.83, 6.24)
	T1	89	6.25	(5.95, 6.55)	140	6.44	(6.22, 6.67)	212	6.54	(6.37, 6.72)
	T2	65	6.23	(5.93, 6.53)	66	6.53	(6.23, 6.83)	143	6.66	(6.45, 6.86)
Calcium-rich food preference score^c^	T0	103	3.39	(3.16, 3.61)	152	3.33	(3.16, 3.5)	212	3.41	(3.25, 3.57)
	T1	88	3.47	(3.24, 3.7)	139	3.33	(3.13, 3.53)	205	3.61	(3.47, 3.76)
	T2	62	3.40	(3.14, 3.67)	65	3.28	(3.01, 3.55)	143	3.55	(3.36, 3.74)
Total calcium intake (mg)^c^	T0	95	919	(818, 1020)	152	1066	(980, 1152)	207	1055	(979, 1131)
	T1	89	916	(809, 1022)	139	1178	(1071, 1286)	207	1194	(1118, 1269)
	T2	61	922	(805, 1038)	65	1125	(987, 1264)	145	1065	(981, 1150)
MET score^c^	T0	110	24.62	(22.52, 26.71)	155	25.08	(23.19, 26.98)	211	24.85	(23.31, 26.39)
	T1	89	27.44	(25.27, 29.61)	140	27.34	(25.42, 29.25)	213	28.89	(27.49, 30.29)
	T2	66	24.94	(21.96, 27.92)	66	25.13	(22.82, 27.44)	143	28.44	(26.66, 30.23)
WBF score^c^	T0	110	3.94	(3.52, 4.36)	155	3.97	(3.62, 4.33)	211	4.00	(3.7, 4.3)
	T1	89	4.27	(3.84, 4.7)	140	4.53	(4.15, 4.91)	213	4.81	(4.53, 5.08)
	T2	66	4.08	(3.5, 4.67)	66	4.14	(3.64, 4.63)	143	4.78	(4.43, 5.13)
Grip strength (kg)	T0	109	10.48	(10.04, 10.91)	154	10.85	(10.47, 11.24)	212	11.05	(10.46, 11.64)
	T1	91	10.92	(10.4, 11.44)	137	11.11	(10.68, 11.54)	215	11.40	(11.02, 11.79)
	T2	61	12.04	(11.28, 12.8)	66	12.75	(12.12, 13.39)	151	12.71	(12.22, 13.21)
Vertical jump (in)	T0	112	11.19	(10.79, 11.59)	152	11.32	(11.01, 11.63)	206	11.46	(11.18, 11.74)
	T1	91	11.55	(11.15, 11.96)	137	11.6	(11.27, 11.93)	214	11.92	(11.63, 12.22)
	T2	62	12.35	(11.82, 12.88)	65	12.26	(11.82, 12.7)	151	12.27	(11.96, 12.58)
Bone stiffness index	T0	50	62.95	(59.15, 66.75)	72	63.39	(61.13, 65.65)	105	64.57	(62.46, 66.69)
	T1	71	64.34	(61.99, 66.69)	106	64.70	(62.55, 66.84)	135	64.95	(63.14, 66.77)
	T2	50	62.64	(60.09, 65.19)	50	65.72	(62.73, 68.71)	124	64.92	(63.25, 66.58)

*Abbreviations*: *BMI*, body mass index, *MET* metabolic equivalent time, *WBF* weight-bearing factor

Range of scores: Calcium-rich food knowledge score: 0–11; Physical activity knowledge score: 0–9; Calcium-rich food preference score: 0–5; Total calcium intake (mg): 0–3661.5; MET score: 0–46.8; WBF score: 0–7.5

^a^Average weight of C is significantly lower than B

### Bone quality and muscular strength

**Fig. 3:**
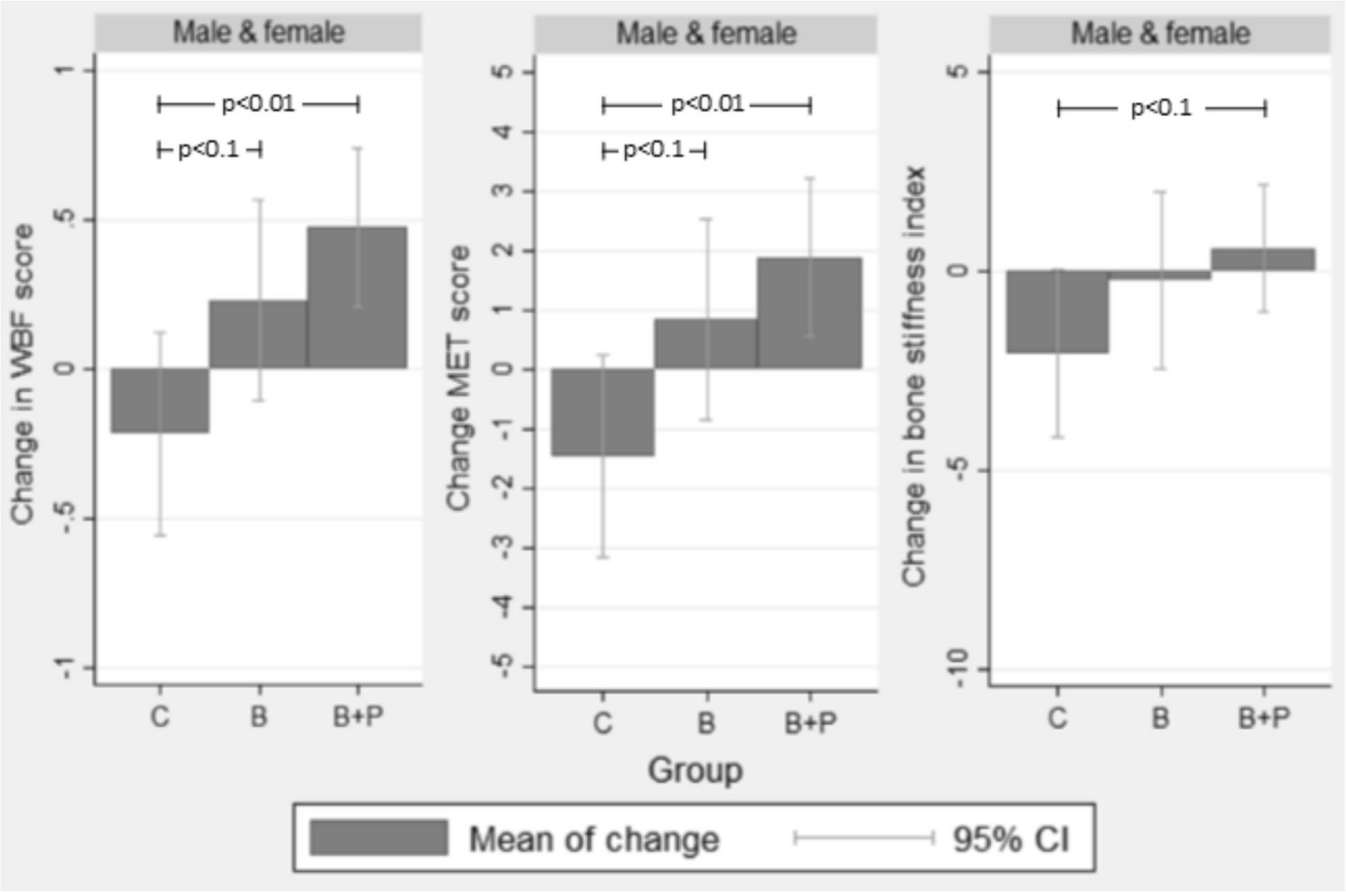
Mean adjusted changes in WBF score, MET score, and bone stiffness by group^a^. Abbreviations: WBF, weight-bearing factor; MET, metabolic equivalent time; C, Control; B, BONES; B + P, BONES + Parent; CI, confidence interval. ^a^Mixed effects regression model adjusted for time point, baseline age, and race/ethnicity (white, black, Hispanic, others); and adjusted for individual nested within after-school program as cluster effects. ^b^Error bars represent 95% confidence intervals derived from standard error. ^c^CIs that do not cross y = 0 indicate the mean change is significantly different from 0

Overall, bone quality data were collected from 35% of male and 46% of female participants. Boys and girls in B + P demonstrated an increase in bone stiffness compared to C (*p* = 0.05 – increase of 0.6 units-per-year in B + P, compared to a reduction of 2.1 units-per-year in C); and when B and B + P were pooled together bone stiffness increased compared to C, although not significantly (*p* = 0.06 – increase of 0.3 units-per-year in B + P, compared to a reduction of 2.1 units-per-year in C) (Fig. 3). When boys and girls were considered separately, boys in all three groups demonstrated a negative rate of change in bone stiffness, while their female counterparts in both intervention groups showed positive rates of change. Although girls alone in neither B nor B + P improved bone stiffness significantly compared to C, in the pooled comparisons, girls in the intervention showed a significant rate of improvement in bone stiffness, over time (*p* < 0.01, increase in 2.1 units-per-year). Grip strength and vertical jump increases were not significantly different among groups; however, boys in B showed a moderate (NS) increase in vertical jump compared to C (p = 0.06).

### Body composition

There was no difference between the rate of change in BMI z-scores across groups and sexes (Table 3). There was a significantly positive rate of increase in percent body fat among all children except boys in C. This resulted in a significant difference for boys in both intervention groups.

**Table 3:** Rates of change in outcomes estimated by mixed effects model by sex and program status

		Rate of change of outcome (per year)								
		Males & Females			Males			Females		
Outcome of each model	Group indicator ^a^	Rate of change (unit/year)	Standard error	*p*-value vs. C^a^	Rate of change (unit/year)	Standard error	*p*-value vs. C^a^	Rate of change (unit/year)	Standard error	*p*-value vs. C^a^
BMI z-score	B + P	0.02322	0.02401	0.1292	0.03198	0.04089	0.1886	0.01316	0.02422	0.4654
	B	0.04661	0.03001	0.3969	0.08685	0.04747	0.6667	0.00321	0.03359	0.3984
	C	0.08362**	0.03161	–	0.11626*	0.04888	–	0.04514	0.03636	–
Percent body fat (%)	B + P	1.3784**	0.4691	0.2319	1.7398**	0.5517	0.0265	1.1805*	0.4845	0.8099
	B	1.8076**	0.5588	0.1021	2.2980***	0.6407	0.0076	1.8399**	0.6219	0.6313
	C	0.4771	0.5882	–	−0.1397	0.6306	–	1.3871	0.7070	–
Calcium-rich food knowledge score	B + P	0.4788***	0.0855	0.9342	0.4306**	0.1425	0.8080	0.4897***	0.1151	0.7887
	B	0.7524***	0.1097	0.0680	0.7004***	0.1660	0.3458	0.7848***	0.1572	0.1333
	C	0.4672***	0.1104	–	0.4827**	0.1593	–	0.4345*	0.1706	–
Physical activity knowledge score	B + P	32.88***	8.27	0.5947	35.96**	12.58	0.5123	29.99**	10.95	0.9289
	B	44.58***	10.56	0.2088	40.46**	15.14	0.4153	48.81**	14.72	0.3449
	C	25.70*	10.64	–	23.43	14.33	–	28.26	15.92	–
Calcium-rich food preference score	B + P	0.1084	0.0619	0.8224	0.1353	0.0938	0.5823	0.0948	0.0835	0.8278
	B	0.1088	0.0795	0.8375	0.0668	0.1115	0.9517	0.1313	0.1144	0.9816
	C	0.0857	0.0800	–	0.0575	0.1053	–	0.1274	0.1240	–
Total calcium intake	B + P	−9.58	42.35	0.4939	−14.39	55.73	0.5383	−22.70	46.75	0.6603
	B	15.62	52.08	0.7695	−33.04	65.98	0.4407	47.57	62.68	0.7301
	C	37.75	54.57	–	37.68	63.32	–	14.79	71.10	–
Trimmed calcium intake (< 2500 for age up to 9, < 3000 for age > =9)	B + P	−6.44	38.28	0.3339	−20.00	50.79	0.2724	−7.26	44.34	0.7929
	B	3.87	47.67	0.4653	−35.06	51.23	0.2377	24.60	59.38	0.9050
	C	54.06	49.38	–	64.94	57.88	–	13.89	67.01	–
MET score	B + P	1.8873**	0.6772	0.0026	1.6754	1.0115	0.0039	2.0405*	0.8800	0.3259
	B	0.8487	0.5874	0.0596	1.7086	1.2161	0.0076	−0.1833	1.1811	0.6910
	C	−1.4592	0.8682	–	−2.8420*	1.1566	–	0.5103	1.2797	–
WBF score	B + P	0.4746***	0.1349	0.0019	0.5125*	0.2052	0.0010	0.4260*	0.1753	0.5584
	B	0.2317	0.1708	0.0679	0.4448	0.2454	0.0046	0.0037	0.2349	0.4753
	C	−0.2141	0.1731	–	− 0.5335*	0.2339	–	0.2445	0.2550	–
Grip strength (kg)	B + P	1.2406***	0.1461	0.6682	1.0568***	0.1802	0.9395	1.3980***	0.1709	0.4730
	B	1.0055***	0.1787	0.6049	1.0356***	0.2122	0.9988	1.0197***	0.2244	0.6379
	C	1.1391***	0.1861	–	1.0361***	0.2050	–	1.1789***	0.2522	–
Vertical jump (in)	B + P	0.6154***	0.1473	0.6493	0.6276***	0.1773	0.8487	0.5810**	0.1747	0.3824
	B	0.2651	0.1780	0.0759	0.0243	0.2089	0.0601	0.5269*	0.2237	0.3414
	C	0.7233***	0.1857	–	0.5761**	0.2027	–	0.8497**	0.2538	–
Bone stiffness	B + P	0.5682	0.8032	0.0506	−1.6887	1.4825	0.4189	2.0487*	0.8608	0.0995
index (3 groups)	B	−0.2319	1.1187	0.2368	−4.0010	2.0555	0.8523	2.0957	1.1986	0.1386
	C	−2.0669	1.0574	–	−3.5066*	1.6495	–	−0.5761	1.3078	–
Bone stiffness	B + P/B	0.3026	0.6527	0.0603	−2.4862*	1.2067	0.6204	2.0757**	0.6990	0.0797
index (2 groups)	C	−2.0670	1.0584	–	−3.5101*	1.6554	–	−0.5754	1.3078	–

*Abbreviations*: *B + P* BONES + Parent, *B* BONES, *C* Control, *BMI* body mass index, *MET* metabolic equivalent time, *WBF* weight-bearing factor

Adjusted for time point, baseline age, and race/ethnicity (white, black, Hispanic, others); adjusted for individual nested within after-school program as cluster effects

In total, 2476 observations were made in the three time points. Numbers of observations used in the above analysis range from 1828 (74%) to 1902 (77%) for all the outcomes except bone stiffness. For bone stiffness, the number of observation used in the analysis is 981 (40%)

^*a*^*p* vs. C: *p*-value of the difference between the slopes of the intervention (B + P or B) and the control group

* *p* < 0.05; ** *p* < 0.01; *** *p* < 0.001; these *p*-values indicate if the rate of change is significantly different from zero

### Knowledge and behavioral outcomes

The predicted annual rates of change are shown in Table 3. In both sexes, all groups increased significantly in their ability to identify calcium-rich foods. Although B had the largest positive change, then B + P, they were not significantly different from C. Knowledge of bone-strengthening activities was also significantly higher in both B and B + P in both sexes at the end of the study, but the rate of increase was not significantly higher than C. There was no change among groups in preference for calcium-rich foods or calcium intake, nor did a further analysis by low calcium intake status at baseline reveal a systematically different pattern of preference among these children than those with higher baseline intake (results not shown). When pooled by sex, children in B + P showed statistically significant increases in MET and WBF scores compared to children in C (*p* < 0.01). Although children in B also showed increases in MET and WBF scores, these were not significantly different from C (*p* < 0.10) (Fig. 3). When stratified by sex, reported MET and WBF scores increased for boys in all three groups, and reported increases for boys in B and B + P were significantly higher than those in C. In girls, only the B + P group showed significant increases in MET and WBF-scores, although they were not significantly different from C.

## Discussion

The BONES Project demonstrated that a community-based intervention among early elementary school children is feasible to implement in diverse, low-income after-school programs. The intervention was effective at improving some bone health behaviors as revealed by findings that children in B + P and boys in both intervention arms (B and B + P) showed statistical improvement in their reported physical activity behaviors. In addition, boys in B had an increase, albeit non-significant, in vertical jump. There was also an increase in bone stiffness compared to controls in the B + P group. These relatively modest, yet encouraging findings in bone stiffness may be attributed to the younger age of children in the BONES Project (6- to 9-yrs) compared to previous bone-building interventions showing improvement. In addition, factors associated with the after-school program setting such as high staff-turnover, time-constraints, and day-to-day variations in child and staff attendance, could have impacted the dose and quality of the intervention. Although there was a positive increase in percent body fat in all groups, except boys in C, this is unlikely to be clinically meaningful given pre-pubescence and a non-significant difference in BMI z-score. Together, the outcomes are encouraging for sustained work in after-school programs to serve as health intervention platforms during early childhood.

Previous interventions targeting bone-building behaviors and osteoporosis prevention in children have focused on schools as a predominant intervention platform with only a few focusing on the after-school program setting. Examples include Daley et al.’s Specialist-led, school Physical Education-based, intervention for 8-year old boys and girls, [13]; the CAPO Kids Trial – a school-based randomized controlled, high intensity interval trial, to enhance bone and reduce fat in girls, ages 10-to-11 [14]; and Meyer et al.’s classroom-based intervention to improve bone BMC and BMD in 1st and 5th grade boys and girls [15], among others. While interventions focused outside of the school environment have incorporated other aspects of bone health including calcium intake and knowledge of bone-building behaviors, these have been largely focused on girls (e.g. Girl Scout meetings [47] and online health behavior change programs for girls [12]).

Findings from the BONES Project support and build upon prior interventions in both reach and intervention design. By expanding on the school-based model and intervening through an after-school program platform, the BONES Project delivered a three-component curriculum – diet, physical activity, and education – to a larger, ethnically diverse, sample of both boys and girls who were younger than in other intervention trials. The benefits and importance of bone-strengthening interventions for children at younger ages are well demonstrated [8, 48].

While community-based approaches are common practices for behavior change interventions targeting children [49], outcomes have been mixed with respect to increases in bone-strengthening behaviors and bone quality; only two had statistically significant increases in total bone area [13, 15]. Although Daley et al. measured physical activity habits and calcium intake, no improvements were observed in either outcome. Neither of the bone-building interventions [12, 47] observed an increase in physical activity metrics and only the one focused on 14–16 year old girls saw an increase in BMD of the Spine and Trochanter. The BONES Project likewise did not find significant improvements in the majority of outcomes. Calcium rich food knowledge for all children improved, although not differentially by intervention assignment. We also did not observe significant increases in calcium intake for either sex. A significant increase in physical activity levels was observed for boys in both intervention groups and all children in B + P compared to controls, but not for girls alone. Additionally, though a statistically significant increase in bone stiffness was not detected, a pooled analysis of both intervention arms revealed that for girls participating in the intervention, the mean change in stiffness was an increase of 2.08 units per year, compared to a drop of 0.58 units-per year for the control (*p* = 0.08). Such an effect could be practically important and may merit further investigation.

### Limitations

Previous research demonstrates the strength of field-based interventions in their ability to connect with the community and have high generalizability [50]; however, a number of limitations are inherent in this research design – particularly in the after-school program setting – that potentially diluted the dose and quality of the intervention. First, the high frequency of staff turnover in the after-school programs required continuous re-training which affected the ability to continuously implement the curriculum. In some programs, staff also varied on a day-to-day basis which may have also limited their ability to implement with fidelity. Second, time constraints and difficulty with machinery such as the calcaneal quantitative ultrasound (QUS) device limited the number of children who could complete bone quality assessments, although every effort was made to measure all children. Third, the variability in child attendance limited the dose of the intervention as not all children attended the after-school program every day. This variation may have also impeded the ability to detect any significant improvement in bone stiffness for the intervention group. Higher attendance at an obesity-prevention intervention in an after-school program setting was previously linked with greater increases in bone-health outcomes [20].

Although a great deal of attention was placed on process evaluation, the personnel in the after-school programs had difficultly tracking and reporting daily attendance which may have further limited our ability to accurately assess dose and intervention fidelity at the individual (child)-level. In addition, although inadequate calcium intake is highly prevalent in the general population, the majority of children in the BONES Project did not appear to have inadequate intake at baseline (data not shown), which may have limited the ability to detect an increase in calcium-intake from the intervention. Lastly, at the time that this study was conducted, there were no assessment tools which adequately captured physical activity levels and calcium intake among young children in the field. Despite extensive work developing and testing new measures for use in the BONES Project [38, 40], these tools may not be sensitive enough to detect change.

### Future considerations

The BONES Project demonstrated that after-school programs may serve as a potential platform for bone-building behavioral interventions for children as others have been successful with health interventions in this environment [51]; however greater intervention intensity may be needed for larger impact. We present a potentially feasible and sustainable model by training large groups of existing after-school program leaders in diverse after-school programs rather than specialist-led initiatives. This platform allows for greater opportunity to make larger-scale environmental modifications, which can improve children’s health behavior and health outcomes including peak bone mass [52]. Future investigators employing the BONES framework should consider user-friendliness of evaluation tools given time constraints of the subjects and program. The quantitative ultrasound device did not work well with children with especially small or narrow feet, and children who did not sit still. Therefore, use of the machinery in the field should be considered, and alternative strategies to increase the percent of participants completing these measurements should be explored. Additionally, while the BONES Project increased availability and accessibility to calcium-rich snacks and physical activity equipment, other environmental change strategies, including those at the policy-level, were not targeted. Exploration of these strategies, as well as further research to understand the cost-effectiveness of intervening through an after-school program, particularly when compared to school-based programs, serve as important next steps in understanding best practices of reaching children with health behavior interventions. Lastly, future research is needed to understand interrelated factors that influence bone health and to assess other indices of bone strength, such as the material and structural properties of bone during growing years.

## Conclusion

The BONES plus parent component of the intervention demonstrated encouraging bone and physical activity outcomes. Community-based interventions conducted in an after-school program-based setting, coupled with parental engagement present a potentially feasible approach for reaching young children to encourage bone-building behaviors that can prevent the onset of osteoporosis in adulthood. The intensity and duration of the program that is needed to significantly impact bone and behavior (diet and physical activity) changes in both boys and girls is still unknown and is likely greater than was anticipated in the BONES Project. Future research should consider cost-effectiveness when delivering programs with the capability for broad reach.

## Data Availability

The datasets generated or analyzed during this study are available from the corresponding author on reasonable request.

## Abbreviations

B + P: BONES Project plus parent outreach intervention group
B: BONES Project intervention group
BMC: Bone mineral content
BMD: Bone mineral density
BMI: Body mass index
BONES: Beat Osteoporosis – Nourish and Exercise Skeletons
BUA: Broadband ultrasound attenuation
C: Control or delayed intervention group
CDC: Center for disease control and prevention
FDA: Food and drug administration
MET: Metabolic equivalent time
QUS: Quantitative ultrasound
SES: Socioeconomic status
SOS: Speed of sound
WBF: Weight-bearing factor
YMCA: Young men’s Christian association
